# Novel CD19-specific γ/δ TCR-T cells in relapsed or refractory diffuse large B-cell lymphoma

**DOI:** 10.1186/s13045-023-01402-y

**Published:** 2023-01-21

**Authors:** Chenggong Li, Fen Zhou, Jing Wang, Qi Chang, Mengyi Du, Wenjing Luo, Yinqiang Zhang, Jia Xu, Lu Tang, Huiwen Jiang, Lin Liu, Haiming Kou, Cong Lu, Danying Liao, Jianghua Wu, Qiuzhe Wei, Sha Ke, Jun Deng, Cheng Liu, Heng Mei, Yu Hu

**Affiliations:** 1 Institute of Hematology, Union Hospital, Tongji Medical College, Huazhong University of Science and Technology, 1277 Jiefang Avenue, Wuhan, 430022 Hubei China; 2 Hubei Clinical Medical Center of Cell Therapy for Neoplastic Disease, Wuhan, 430022 China; 3 Department of Pediatrics, Union Hospital, Tongji Medical College,, Huazhong University of Science and Technology, Wuhan, 430022 China; 4 Department of Radiology, Union Hospital, Tongji Medical College, Huazhong University of Science and Technology, Wuhan, 430022 China; 5 Eureka Therapeutics, Inc, Emeryville, CA 94608 USA

**Keywords:** γ/δ TCR-T cells, DLBCL, Primary CNS lymphoma, Relapsed or refractory, Cellular immunotherapy

## Abstract

**Background:**

T cell receptor (TCR)-T cells possess similar effector function, but milder and more durable signal activation compared with chimeric antigen receptor-T cells. TCR-T cell therapy is another active field of cellular immunotherapy for cancer.

**Methods:**

We previously developed a human anti-CD19 antibody (ET190L1) and generated novel CD19-specific γ/δ TCR-T cells, ET019003, by fusing the Fab fragment of ET190L1 with γ/δ TCR constant chain plus adding an ET190L1-scFv/CD28 co-stimulatory molecule. ET019003 cells were tested in preclinical studies followed by a phase 1 clinical trial.

**Results:**

ET019003 cells produced less cytokines but retained comparable antitumor potency than ET190L1-CAR-T cells in vivo and in vitro. In the first-in-human trial, eight patients with relapsed or refractory DLBCL were treated. CRS of grade 1 was observed in three (37.5%) patients; ICANS of grade 3 was noted in one (12.5%) patient. Elevation of serum cytokines after ET019003 infusion was almost modest. With a median follow-up of 34 (range 6–38) months, seven (87.5%) patients attained clinical responses and six (75%) achieved complete responses (CR). OS, PFS and DOR at 3 years were 75.0%, 62.5%, and 71.4%, respectively. Notably, patient 1 with primary CNS lymphoma did not experience CRS or ICANS and got an ongoing CR for over 3 years after infusion, with detectable ET019003 cells in CSF. ET019003 showed striking in vivo expansion and persisted in 50% of patients at 12 months. Three patients received a second infusion, one for consolidation therapy after CR and two for salvage therapy after disease progression, but no response was observed. ET019003 expansion was striking in the first infusion, but poor in the second infusion.

**Conclusions:**

CD19-specific γ/δ TCR-T cells, ET019003, had a good safety profile and could induce rapid responses and durable CR in patients with relapsed or refractory DLBCL, even primary CNS lymphoma, presenting a novel and potent therapeutic option for these patients.

*Trial registration*: NCT04014894.

**Supplementary Information:**

The online version contains supplementary material available at 10.1186/s13045-023-01402-y.

## Introduction

Non-Hodgkin lymphoma (NHL) is the most common hematological malignance, and diffuse large B-cell lymphoma (DLBCL) is the most common subtype, accounting for 30–40% of newly diagnosed cases worldwide [1, 2]. In the rituximab era, over 60% can be cured with rituximab-based regimens, but patients who have failed after the frontline therapy have a poor prognosis [3]. The overall response rate (ORR) to the second-line treatment was 44% with a median overall survival (OS) of 8 months [4]. The ORR to the third-line chemotherapy was 39% with a median OS of 4.4 months [5]. A patient-level pooled SCHOLAR-1 study indicated the ORR to the next-line therapy of 26% and the median OS of 6.3 months for patients with refractory DLBCL [6]. Besides, primary central nervous system lymphoma (PCNSL) still poses a challenge [7].

CD19-specific chimeric antigen receptor (CAR)-T cells have showed remarkable efficacy in NHL, and three cell products are currently approved by the Food and Drug Administration in the treatment of relapsed or refractory (RR) DLBCL: axicabtagene ciloleucel (axi-cel), tisagenlecleucel (tisa-cel), and lisocabtagene maraleucel (liso-cel). However, cytokine release syndrome (CRS) and immune cell-associated neurotoxicity syndrome (ICANS) are clinical challenges that remain and can be mitigated further by other therapeutic approaches. T cell receptor (TCR)-engineered T cell therapy, by replacing the antigen recognition domain of TCR with an antibody-derived recognition fragment, is another active field of cellular immunotherapy for cancer. TCR-T cells can be used to target either peptide-major histocompatibility complexes or cell-surface antigens by using TCR-mimic or conventional antibodies, respectively. TCR-T cells possess milder and more durable signal activation, but similar effector function compared with CAR-T cells [8]. A head-to-head comparison demonstrates that activation through TCR versus CAR that uses the same recognition domains has 10–100-fold higher antigen sensitivity and induces dramatically less cytokine release [9]. We previously developed a human anti-CD19 antibody (ET190L1) and found that ET190L1-TCR-T cells maintained comparable antitumor potency with less cytokine release to CD28-costimulated ET190L1-CAR-T and CD137-costimulated CTL019 cells [10]. ET019003 cells are novel anti-CD19 γ/δ TCR-T cells generated based on ET190L1-TCR-T cells by adding a single-chain fragment variable (scFv)/CD28 co-stimulatory molecule for further boosting T cell activation [11]. Here, we report outcomes for adult patients with RR DLBCL treated with ET019003 cells.

## Material and methods

### ET019003 cell production

The fully human anti-CD19 antibody (ET190L1) was selected from Eureka Therapeutics E-ALPHA® phage display library, and the characterization of ET190L1 was identified as previously described [10]. ET190L1-TCR fuses the Fab fragment of ET190L1 with the C-terminal signaling domain of a γ/δ TCR, which is derived from the human TCR γ constant chain (UniProtKB—P0CF51) and δ constant chain (UniProtKB—B7Z8K6). The heavy chain domain of the Fab fragment is fused to the δ chain, and the light chain domain is fused to the γ chain. Moreover, an independent chimeric molecule consisting of the scFv of ET190L1 and the CD28 transmembrane and intracellular domain is added to further promote T cell activation [11]. The ET190L1-TCR and the scFv/CD28 co-stimulatory molecule sequences were cloned into a pCDH lentiviral vector (Systems Biosciences, California, USA) for generation of ET019003 cells. The preclinical studies, production, and monitoring of ET019003 cells are detailed in the supplemental methods (Additional file 1).

### Trial design and patients

The first-in-human, single-center, phase 1 study (ClinicalTrials.gov identifier NCT04014894) was designed to evaluate the safety and efficacy of ET019003 cells in patients with CD19^+^ malignancies, of whom eight with RR DLBCL are reported here. The inclusion criteria are provided in Supplemental Methods (Additional file 1). The study was approved by the Medical Ethics Committee of the Union Hospital affiliated to Huazhong University of Science and Technology, Wuhan, China. All patients provided written informed consent.

Bridging therapy was not allowed between leukapheresis and infusion. The preconditioning regimens consisted of cyclophosphamide 250 mg/m^2^ on day − 5 and fludarabine 25 mg/m^2^ on day − 5 to − 3. On day 0, the patients received an intravenous infusion of ET019003 cells at the planned dose levels of 2 × 10^6^ and 4 × 10^6^ TCR + T cells/kg with a standard 3 + 3 design. If the patient did not experience ≥ grade 3 adverse events (AEs) excluding hematological toxicities within 1 month after infusion, repeated infusions at the same dose level were allowed. If the patient did not achieve a complete response (CR) with response assessed at month 3, a second infusion was allowed at the same or a higher dose level.

### End points

The primary objectives were incidence of AEs and ORR. CRS and ICANS were graded using the ASTCT consensus grading [12]. All other AEs were graded according to CTCAE, version 5.0. Dose-limiting toxicities (DLTs) were defined as ET019003-related AEs within 30 days after infusion and included ≥ grade 3 cardiac, hepatic, pulmonary, and renal toxicities, ≥ grade 3 CRS and ICANS that lasted over 72 h after treatment. Exceptions to this definition were not counted as a DLT. Response was assessed using the Lugano criteria [13]. The secondary objectives included duration of response (DOR), progression-free survival (PFS), OS, expansion and persistence of ET019003 cells, and serum cytokines in the PB after infusion. DOR, PFS, and OS were defined per the revised response criteria for malignant lymphoma [14]. Serum cytokines were measured by cytometric beads array using the commercial kits as previously used [15]. Only the first infusion was included in the main analyses of safety and efficacy. Exploratory endpoints included the safety and efficacy among patients retreated with ET019003 cells.

### Imaging and pathological examination

^18^F-fluorodeoxyglucose positron emission tomography-computed tomography (PET-CT), CT, magnetic resonance imaging, cerebrospinal fluid (CSF) assessment, and biopsy were performed following the Lugano criteria [13]. The pathologic and immunohistochemical assessments of tumor tissue were conducted and reviewed by two independent pathologists.

### Statistical analyses

All eight patients who received the infusion were included in the analyses. Descriptive statistics include means with 95% confidence interval (CI) or medians with minimum and maximum (range) for continuous variables and counts and percentages for categorical variables. Missing data were not imputed. Continuous variables were compared using paired t test when the data were normally distributed. Otherwise, the Wilcoxon test was used. DOR, PFS, OS, and associated 95% CI were determined by the Kaplan–Meier methods and compared with the log-rank test between subgroups. Analysis was performed using Graphpad Prism version 8.0. P values less than 0.05 (two-tailed) were considered significant.

## Results

### Design and characteristics of ET019003 cells

**Fig. 1:**
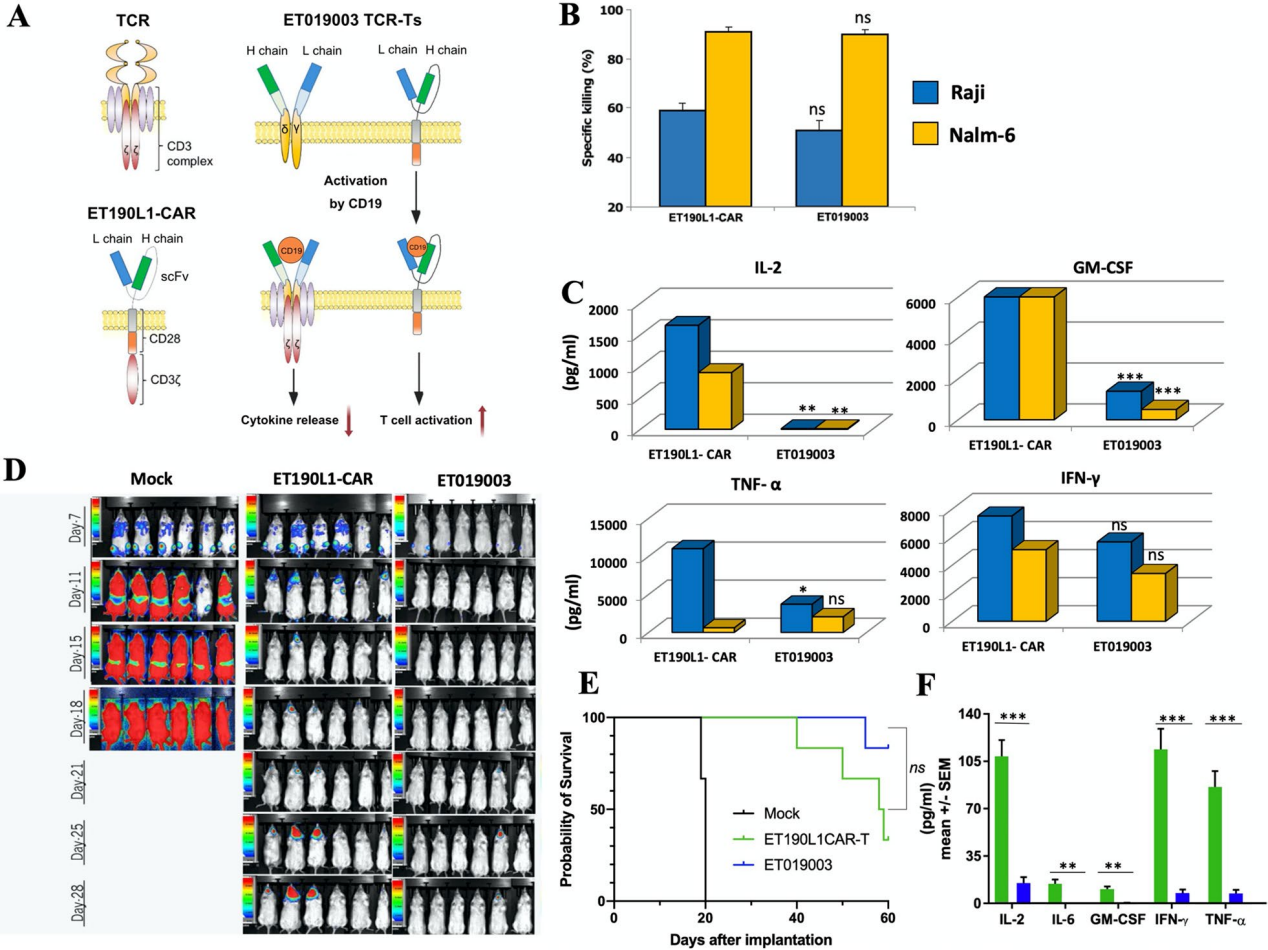
Molecular design and preclinical evaluation of ET019003. **A** Schematic structure of ET019003 compared with natural γ/δ TCR and ET019L1-CAR. L, light; H, heavy; scFv, single-chain fragment variable. **B** Cytotoxicity of ET019003 and ET019L1-CAR-T cells on CD19^+^ Raji and Nalm-6 cells (*n* = 5). **C** Production of interleukin (IL)-2, granulocyte macrophage colony-stimulating factor (GM-CSF), tumor necrosis factor-α (TNF-α), interferon-γ (IFN-γ) in coculture of ET019L1-CAR-T and ET019003 cells with Raji and Nalm-6 cells (*n* = 5). **D** Luciferase live imaging of Raji xenograft mice on different timepoint after receiving mock T cells, ET019L1-CAR-T cells, and ET019003 cells. **E** Kaplan–Meier survival plot of Raji xenograft mice. **F** Serum IL-2, IL-6, GM-CSF, TNF-α, and IFN-γ in Raji xenograft mice on day 7 after infusion of ET019L1-CAR-T and ET019003 cells (*n* = 6). The unpaired t test was used in Fig. **A**–**C** and **F**, and the log-rank test was used in Fig. **E**. Ns, not significant; *P < 0.05; **P < 0.01; ***P < 0.001

ET019003 cells simultaneously expressed fully human ET190L1-TCRs and scFv/CD28 co-stimulatory molecules (Fig. 1A). ET019003 TCR-T cells maintained comparable antitumor potency but released less inflammatory cytokines in vitro and in vivo compared with ET190L1-CAR-T cells (Fig. 1B–F). ET019003 cell manufacturing was successful for all the eight treated patients (6 at 2 × 10^6^/kg, and 2 at 4 × 10^6^/kg). Three patients received a second infusion, the cryopreserved ET019003 cells of the first production was used for patient 5, and a second production was used for patient 2 and 7 (Additional file 1: Table S1). The mean transduction efficiency was 59.52% (range, 44.90–75.90%), with 61.38% of TCR^+^CD4^+^ T cells and 34.93% of TCR^+^CD8^+^ T cells (*n* = 10). The mean turnaround time from leukapheresis to infusion was 14 (range, 11–25) days.

### Patient and treatment characteristics

As of August 20, 2022, the median follow-up time after infusion was 34 (range, 6–38) months. Patient enrollment was ceased in June 2020. Of the 11 patients with DLBCL screened, ten patients were enrolled, and eight patients received ET019003. Two patients died from disease progression and discontinued the study, one died before leukapheresis and another before infusion (Additional file 1: Fig. S1). Patients ranged from 33 to 71 years of age and had received a median of 4 (range, 2–8) prior lines of treatment, and three (37.5%) patients received prior PD-1 inhibitors (Table 1). Five (62.5%) patients had refractory diseases, and three (37.5%) experienced relapses. Patient 1 had PCNSL. Four (50%) patients were germinal center B-cell (GCB) subtype and four (50%) had non-GCB per the Hans algorithm. MYC/BCL2/BCL6 triple expression was detected in four patients (50%), and double expression in two (25%) patients. Five (62.5%) patients had stage IV disease using Ann Arbor staging. Five patients (62.5%) were assigned to the low-intermediate risk group and three (37.5%) were included in high-intermediate risk group per IPI score, respectively.

**Table 1:** Baseline and treatment characteristics of patients

Patient ID	Sex	Age	ECOG	Disease pathology	BCL6/BCL2/C-Myc expression	Prior lines of therapy and most recent response	Prior lines of therapy	Site of diseases	Ann Arbor staging	IPI scores	LDH **(U**/L)	β2 microglobulin (mg/L)
1	F	50	3	DLBCL, non-GCB	No	Radiotherapy, PD	8	Primary CNS lymphomas (right posterior margin of corpus callosum)	IEa	2	363	6.10
						R + HD-MTX + TMZ, PR and then PD						
						Ibutinib, PD						
						Thalidomide, SD						
						PD-1 inhibitor + pemetrexed, PD						
						PD-1 inhibitor* + pemetrexed + carboplatin, PD						
						Daunorubicin + lenalidomide + tcmozolomidc, PD						
						Etoposide + ifosfamide, SD						
2	F	55	2	DLBCL, ABC, NOS	BCL6/BCL2/C-Myc	R-CDOP, PR	5	Mainly in the thoracic cavity, the abdominal cavity, the pelvic cavity and the right thigh	IVa	3	400	3.00
						R-EPCOH, SD						
						R-ICE + lenalidomide, PR and then PD						
						ICE, PR and then PD						
						R-ICE, PR and then PD						
3	F	54	1	DLBCL, GCB	No	Mastectomy	4	Mainly in the right eyeball, and the pelvic cavity	IVb	3	317	4.00
						R-CODOX, CR and then relapse						
						Radiotherapy, PD						
						PD-1 inhibitor* + GVD, relapse						
4	M	40	1	DLBCL, GCB	BCL6/BCL2	R-CHOP, CR and then relapse	4	In the area II of right neck, and the pelvic cavity	IVa	2	183	2.70
						PD-1 inhibitor + GVD, PR and then SD						
						PD-1 inhibitor + ICE, PD						
						PD-1 inhibitor* + paclitaxel, PD						
5	M	71	1	DLBCL, ABC, NOS	BCL6/C-Myc	R-CHOP,CR	4	In the hepatogastric space-pancreatic head	IIEa	2	200	4.80
						R + CVD, NA						
						R maintenance, relapse						
						Gemox, PD						
						R maintenance, PD						
6	F	33	1	DLBCL, GCB, NOS	BCL6/BCL2/C-Myc	R-CHO.NA	6	Mainly in the abdominal cavity	IVa	2	128	4.80
						R-CNO,NA						
						R-GDP,PD						
						R2-GDP, NA						
						R2-GDP + etoposide, PR						
						R-CHOP, PD						
7	F	42	2	DLBCL, ABC, NOS	BCL6/BCL2/C-Myc	R-CHOP, PR and then PD	2	Mainly in the thoracic cavity and the abdominal cavity	IVa	3	1197	1.50
						R2-ICE, PR and then PD						
8	M	52	1	DLBCL, GCB, NOS	BCL67BCL2/C-Myc	R-CHOP, PD	3	In the left heart diaphragm and retroperitoneal space	IIIEb	2	224	3.10
						R + DA-EPOCH, PD						
						R-GDP, PD						

*The duration between last PD-1 inhibitor and date of infusion was 161 days for patient 12, 14 days for patient 3, and 41 days for patient 4. DLBCL: diffuse large B-cell lymphoma; GCB: germinal center B-cell; ABC: activated B-cell; NOS: not otherwise specified; PD: progressive disease; PR: partial response; SD: stable disease; CR: complete response; NA: not available; R: rituximab; HD-MTX: high-dose methotrexate; TMZ: temozolomide; CDOP: cyclophosphamide, liposomal doxorubicin, vincristine, prednisone; EPCOH: etoposide, doxorubicin, vincristine, cyclophosphamide, prednisone; ICE:ifosfamide, carboplatin, etoposide; CODOX: cyclophosphamide, adriamycin, vincristine, intrathecal injection of methotrexate, cytarabine; GVD: gemcitabine, doxorubicin liposome, vinorelbine; CHOP: cyclophosphamide, adriamycin, vincristine, prednisone; CVD: cyclophosphamide, vinoresin, dexamethasone; Gemox: gemcitabine, oxaliplatin; CHO: cyclophosphamide, epirubicin, vinoresin; CNO: cyclophosphamide, mitoxantrone, vinoresin; GDP:Gemcitabine, cisplatin, dexamethasone; R2: rituximab, lenalidomide; DA: dose-adjusted; LDH: lactate dehydrogenase. LDH normally ranges from 109 to 245 U/L and β2 microglobulin from 1.0 to 3.0 mg/L

### Safety

All the eight patients had AEs of grade 3 or higher (Table 2). Three patients (37.5%) experienced grade 1 CRS that resolved spontaneously, with a median onset of 4 (range, 2–9) days and a median duration of 3 (range, 1–8) days. Patient 2 developed grade 3 ICANS, manifested as confusion, barylalia, tremor, and agitation, which occurred after CRS and responded to corticosteroids. ICANS occurred on day 9 after infusion, lasted for 9 days, and was thus judged as a DLT. Consequently, another four patients were treated at the dose of 2 × 10^6^ TCR + T cells/kg. Apart from patient 2, DLTs were not observed in the patient cohort (Additional file 1: Fig. S2). Tocilizumab was not administered. Patient 8 had pulmonary infection on day 15, which lasted for 4 days after antibiotic treatment. Other infectious complications were not observed within 1 month, which was possible due to administrating antiviral and antifungal prophylaxis in these patients. Patient 4 had lymphoma involvement in the intestinal tract, and he suffered acute intestine perforation, and received an emergency surgery on day 16 after infusion. The surgical pathology reconfirmed lymphoma infiltration. All the acute AEs were reversible with supportive treatment.

**Table 2:** Acute and long-term toxicities after ET019003 treatment

Patient ID	Dosage of infused TCR + T cells	CRS (duration)	ICANS (duration)	Treatment for CRS/ICANS	Hematologic AEs (duration)	Related to FC	Other AEs
1	2 × l0^6^/kg	No	No	No	Grade 4 WBC count decreased (d0–dl4) Grade 4 neutrophil count decreased (d0–dl4)	Yes	Viral encephalitis (ml 8) MOG + encephalomyelitis (m30)
2*	1st: 2 × l0^6^/kg	Grade 1 (d2–d8)	Grade 3 (d9–dl4)	Dexamethasone 30 mg	Grade 4 WBC count decreased (dl2–dl9) Grade 4 neutrophil count decreased (d9–dl9) Grade 4 platelet count decreased (dl5-NR)	No	Grade 1 ALT increased (dl3) Grade 2 AST increased (dl3) Grade 1 total bilirubin increased (d28) Grade 1 direct bilirubin increased (d28) Grade 2 hypoalbuminemia (d4-dl 3)
	2nd: 2 × l0^6^/kg	No	No	No	Grade 4 WBC count decreased (d0-NR) Grade 4 neutrophil count decreased (d0-NR) Grade 4 platelet count decreased (d0-NR) Grade 3 anemia (d0-NR)	Yes	Grade 1 total bilirubin increased (d29) Grade 1 direct bilirubin increased (d4-d7, d21-d30) Grade 1 ALT increased (d29–d30) Grade 2 AST increased (d29) Grade 2 hypoalbuminemia (d0–dl6)
3	2 × l0^6^/kg	Grade 1 (d9)	No	No	Grade 3 neutrophil count decreased (d10–d13)	No	Grade 2 ALT increased (m2–m3) Grade 1 AST increased (dl5–m3) Grade 1 ALP increased (m6)
4	2 × l0^6^/kg	No	No	No	Grade 2 neutrophil count decreased Grade 1 total bilirubin increased (d18)	No	Grade 4 intestine perforation (d16) Grade 1 total bilirubin increased (m6) Grade 1 ALT increased (m6–m9) Grade 1 AST increased (m6–m9) Grade 2 hypoalbuminemia (d17–d18)
5	1st: 2 × l0^6^/kg	No	No	No	Grade 3 WBC count decreased (d1–d4) Grade 3 neutrophil count decreased (d4–d8) Grade 3 platelet count decreased (d23–d24)	Yes	Grade 1 total bilirubin increased (m9, ml8, m24) Grade 1 direct bilirubin increased (m9, m18, m24) Grade 2 hypoalbuminemia (d0–d6)
	2nd: 2 × l0^6^/kg	No	No	No	Grade 3 WBC count decreased (d10–d22) Grade 4 neutrophil count decreased (d10–d22) Grade 3 platelet count decreased (d3–1d10)	No	Grade 1 total bilirubin increased (d10–d20) Grade 1 direct bilirubin increased (d8–15)
6	2 × l0^6^/kg	No	No	No	Grade 4 WBC count decreased (d1–d25) Grade 4 neutrophil count decreased (d1–d24) Grade 2 platelet count decreased	Yes	No
7	1st: 4 × l0^6^/kg	Grade 1 (d2)	No	No	Grade 4 WBC count decreased (d0–d10) Grade 4 neutrophil count decreased (d0–d12) Grade 1 anemia	Yes	Grade 1 total bilirubin increased (m6) Grade 1 direct bilirubin increased (m6)
	2nd: 4 × l0^6^/kg	No	No	No	No	NA	No
8	4 × l0^6^/kg	No	No	No	Grade 3 WBC count decreased (d0–d14) Grade 3 neutrophil count decreased (d2–d14) Grade 1 platelet count decreased	Yes	Grade 3 pulmonary infection (d15–d18)

*Patient 2 were followed up to 93 days after the first infusion and 20 days after the second infusion. Severe hematologic toxicities did not recover at the last follow-up session. NR: not recover; NA: not applicable; WBC: white blood cell; d: day; m: month

**Fig. 2:**
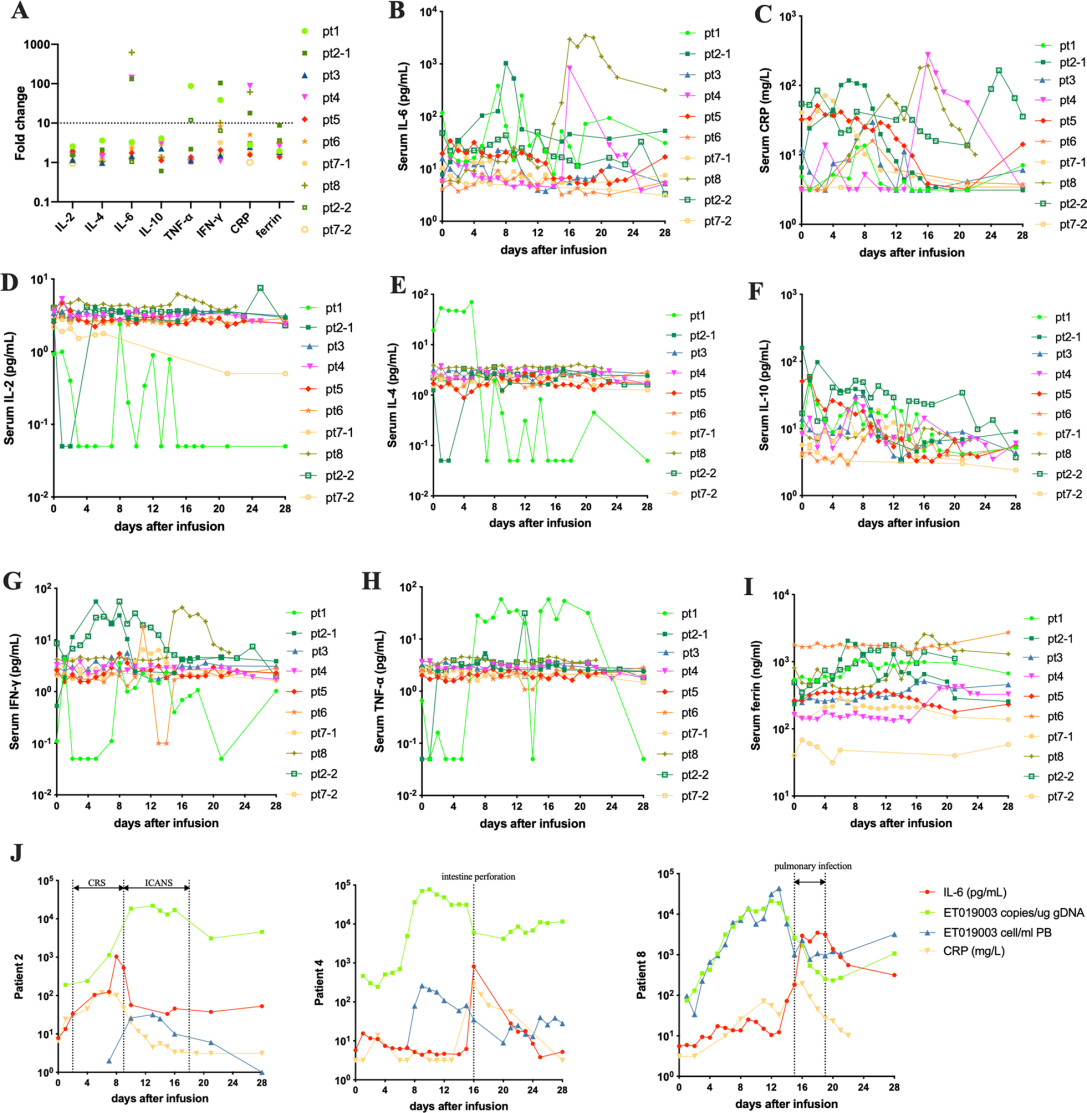
Changes in serum inflammatory markers within 1 month after ET019003 infusion. **A** Fold changes of inflammatory cytokines from baseline to peak (*n* = 10). Patient 5 received the repeated infusions in the outpatient department and data were not available. **B–I** Changes in the serum interleukin (IL)-6, C-reactive protein (CRP), IL-2, IL-4, IL-10, interferon-γ (IFN-γ), tumor necrosis factor-α (TNF-α) and ferritin in individuals. **J** Changes in serum IL-6, CRP, and ET019003 counts and copies in peripheral blood (PB) of patient 2, 4 and 8

The increasing folds of inflammatory cytokines from baseline to peak were modest, except for the elevated serum interleukin (IL)-6 greater than tenfold of the baseline value was observed in patient 2, 4 and 8 (Fig. 2A–I). The elevation of serum IL-6 levels generally coincided in serum C-reaction protein levels and was concurrent with the onset of CRS and ICANS in patient 2, intestine perforation in patient 4, and pulmonary infection in patient 8 (Fig. 2J). Therefore, in addition to ET019003 treatment, there might be alternative etiologies for the elevated inflammatory markers in these three patients.

Hematologic toxicities were the most common AEs, including neutropenia, leukopenia, and thrombocytopenia of grade 3 or 4 in seven (87.5%), six (75%), and two (25%) patients after ET019003 infusion, respectively (Table 2; Additional file 1: Fig. S3a–f). Severe anemia was not observed. The preconditioning regimens exhibited significant adverse effects on leucocytes, lymphocytes, monocytes, and hemoglobin levels, but not on platelets and neutrophils (Additional file 1: Fig. S3g). The median time from infusion to recovery of ≤ grade 2 neutropenia and leukopenia was 13 (range, 4–26) days and 13 (range, 4–26) days, respectively. Delayed recovery from severe thrombocytopenia was observed in patient 2 for over 2 months.

B-cell aplasia, defined as CD19^+^ B-cells representing < 3% of lymphocytes in peripheral blood (PB) [16], was observed in all patients (100%) at baseline (Additional file 1: Fig. S4a). The preconditioning chemotherapy exhibited significant inhibition on T and NK cells in the PB, and ET019003 cells showed effects on T cells (Additional file 1: Fig. S4b–c). CD4^+^ T and CD8^+^ T cells decreased significantly after the preconditioning chemotherapy and expanded remarkably on day 14 after ET019003 infusion with an invert CD4/CD8 ratio (Additional file 1: Fig. S4d–f). Three patients (37.5%) had preexisting hypogammaglobulinemia, defined as serum IgG < 800 mg/dL, IgM < 50 mg/dL, and IgA < 100 mg/dL [17]. The reduction of serum IgG, IgA, and IgM after ET019003 infusion was observed in seven (87.5%), eight (100%) and six (75%) patients, respectively. The recovery of serum IgG, IgA, and IgM to their normal levels during follow-up was observed in three (42.8%), two (25%), and four (66.7%) patients, respectively (Additional file 1: Fig. S4g–i).

Other long-term AEs included viral encephalitis at month 18 and MOG + encephalomyelitis at month 30 of patient 1, and both were treatable. The diagnosis and treatment were detailed in the supplemental results (Additional file 1). ET019003 cells were undetectable at that time in the patient; thus, we supposed that the two delayed AEs were not directly ET019003 cell-mediated.

### Efficacy

**Fig. 3:**
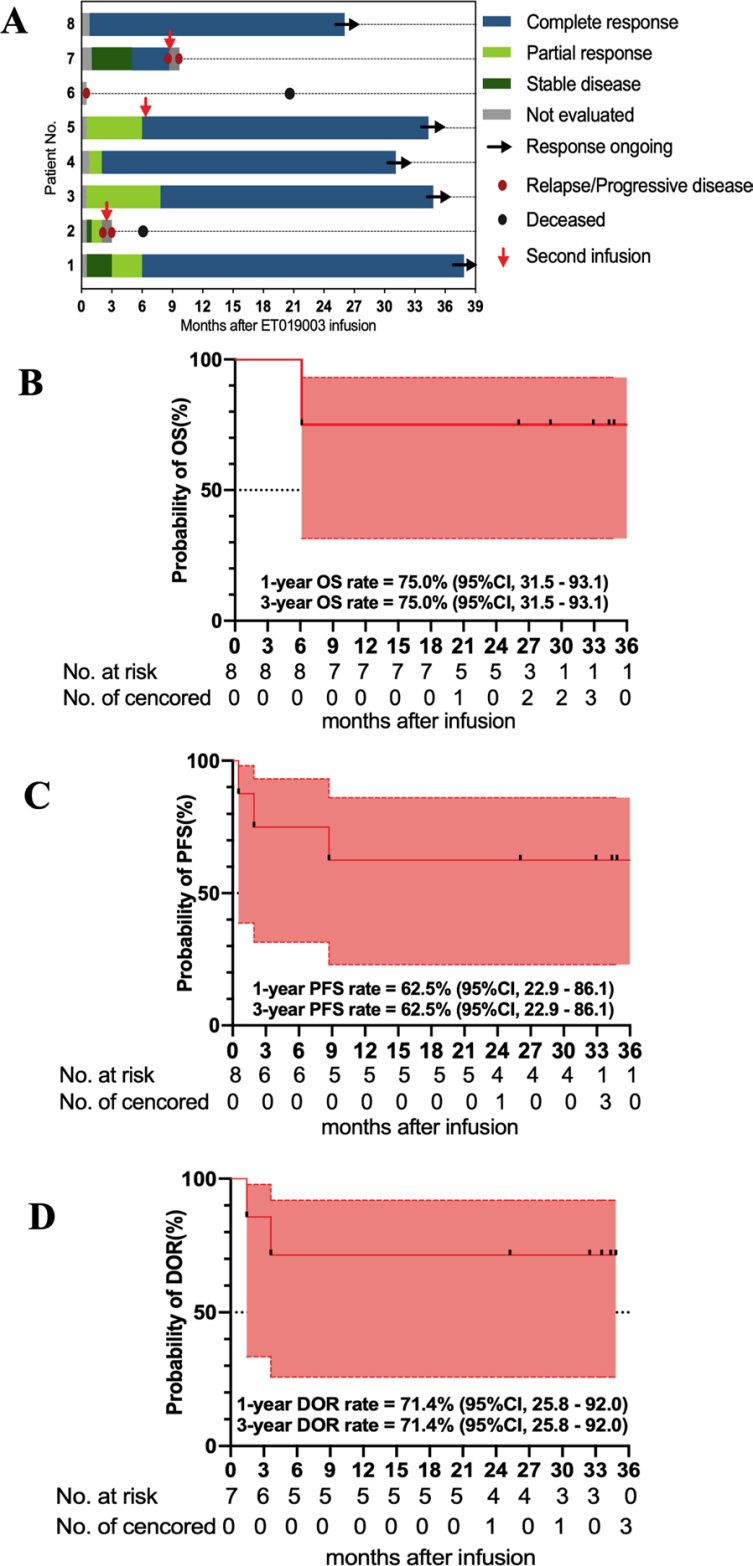
Swimmer’s plot and long-term outcomes of the treated patients. **A** Swimmer’s plot of the eight treated patients. **B–D** Kaplan–Meier estimates of the overall survival (OS), progression-free survival (PFS), and duration of response (DOR)

In the phase 1 trial, seven (87.5%) patients attained clinical responses, six (75%) achieved CR, and five (62.5%) had ongoing CR (Fig. 3A). The Kaplan–Meier estimated OS at 12–36 months were 75.0% (95% CI, 31.5–93.1) (Fig. 3B). The Kaplan–Meier estimated PFS at 12–36 months were 62.5% (95% CI, 22.9–86.1) (Fig. 3C), with DOR at 12–36 months of 71.4% (95% CI, 25.8–92.0) (Fig. 3D).

**Fig. 4:**
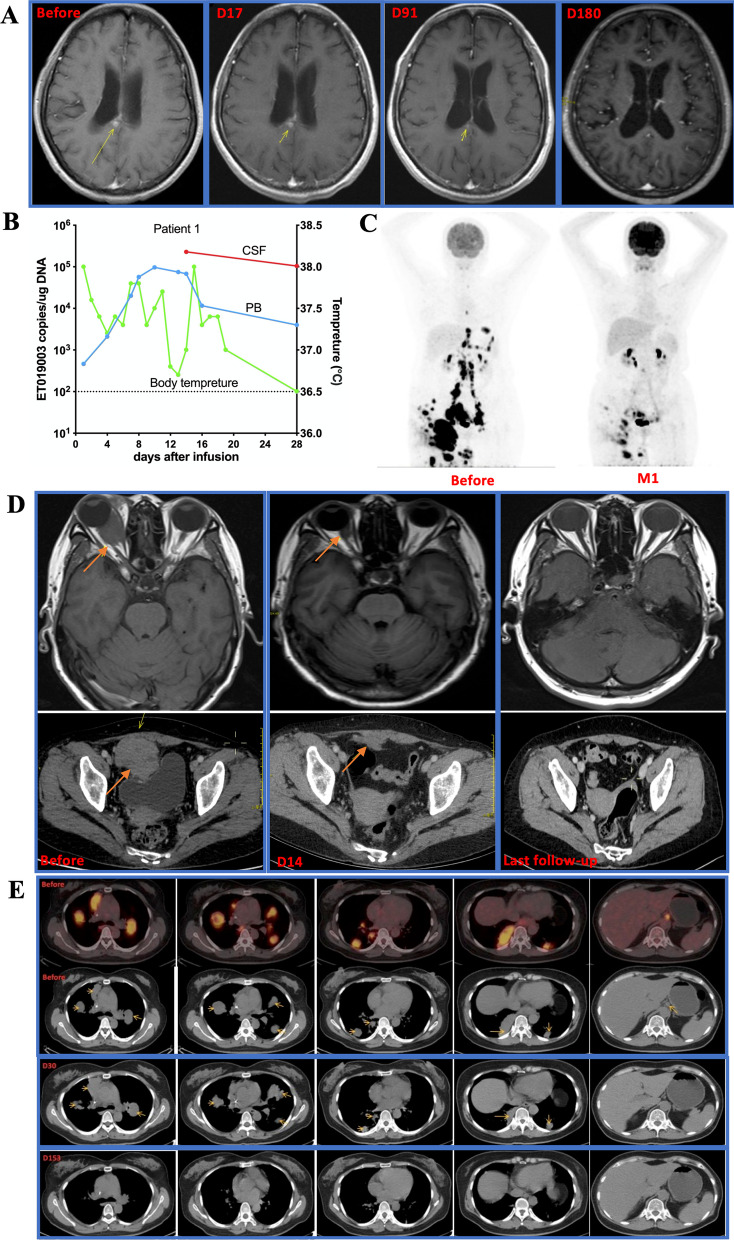
Clinical responses of ET019003 cells. **A** Changes in cranial magnetic resonance imaging (MRI) scans of patient 1. **B** ET019003 copies per microgram (µg) of genomic DNA in peripheral blood (PB) and cerebrospinal fluid (CSF) and body temperature changes in patient 1 within 1 month after infusion. **C** Changes in positron emission tomography-computed tomography (PET-CT) scans of patient 2. **D** Changes in ocular enhanced MRI and abdominal-enhanced CT of patient 3. **E** Changes in PET-CT scans of patient 7

Patient 1 with PCNSL was refractory to previous 8 lines of therapies. She got a continuing CR for over 3 years after ET019003 infusion (Fig. 4A). Numerous ET019003 cells were detectable in both the PB and CSF after infusion (Fig. 4B), indicating that ET019003 cells could sufficiently traffic from the periphery to the CNS. Patient 2 had extensive lesions and attained a quick partial response (PR) at month 1 (Fig. 4C), but the diseases progressed at month 2 (Additional file 1: Fig. S5a). A second tissue biopsy demonstrated DLBCL with expression of CD19, BCL2, C-Myc, P53, and Ki67 (LI: 90%). The patient received a second infusion with poor expansion, and the diseases progressed on day 14; consequently, the patient withdrew from the study for other salvage therapy. Patient 3 had two major lesions in the right eyeball and the pelvic cavity, and she attained a PR on day 14 and an ongoing CR for over 2 years (Fig. 4D). Patient 4 achieved a CR at month 2 and kept CR for over 2 years (Additional file 1: Fig. S5b). ET019003 cells exhibited rapid clearance and durable control of a bulky tumor (8.1 × 6.6 × 7.0 cm, SUVmax 9.4–12.7) in patient 5 (Additional file 1: Fig. S5c). Patient 6 with lymphoma mainly in the abdominal cavity did not respond to ET019003 treatment and withdrew from the study at month 2 (Additional file 1: Fig. S5d). Patient 7 had extensive lesions mainly in the lung and the abdominal cavity and attained a CR at month 5 (Fig. 4E). However, new lesions appeared at month 9, and a second infusion failed to work (Additional file 1: Fig. S5e). Patient 8 had two lymph node lesions in the left heart diaphragm angle and retroperitoneal space, and got a CR on day 24, and kept durable CR at month 24 (Additional file 1: Fig. S5f).

### Secondary infusion

Three patients received a second infusion. Patient 2 and 7 for salvage therapy after disease progression, but response was not observed. Patient 5 kept CR per the Lugano criteria at month 6, but PET-CT scans showed minimal residual lesions in the hepatogastric space-pancreatic head. Despite low levels of ET019003 cells in his PB, we decided to give the patient a repeated infusion in the outpatient department without the preconditioning chemotherapy. He experienced severe neutropenia, leukopenia, and thrombocytopenia, which were self-limiting. Apart from hematologic toxicities observed in patient 2 and 5, other AEs were not observed (Additional file 1: Fig. S6).

### Expansion and persistence of ET019003 cells

**Fig. 5:**
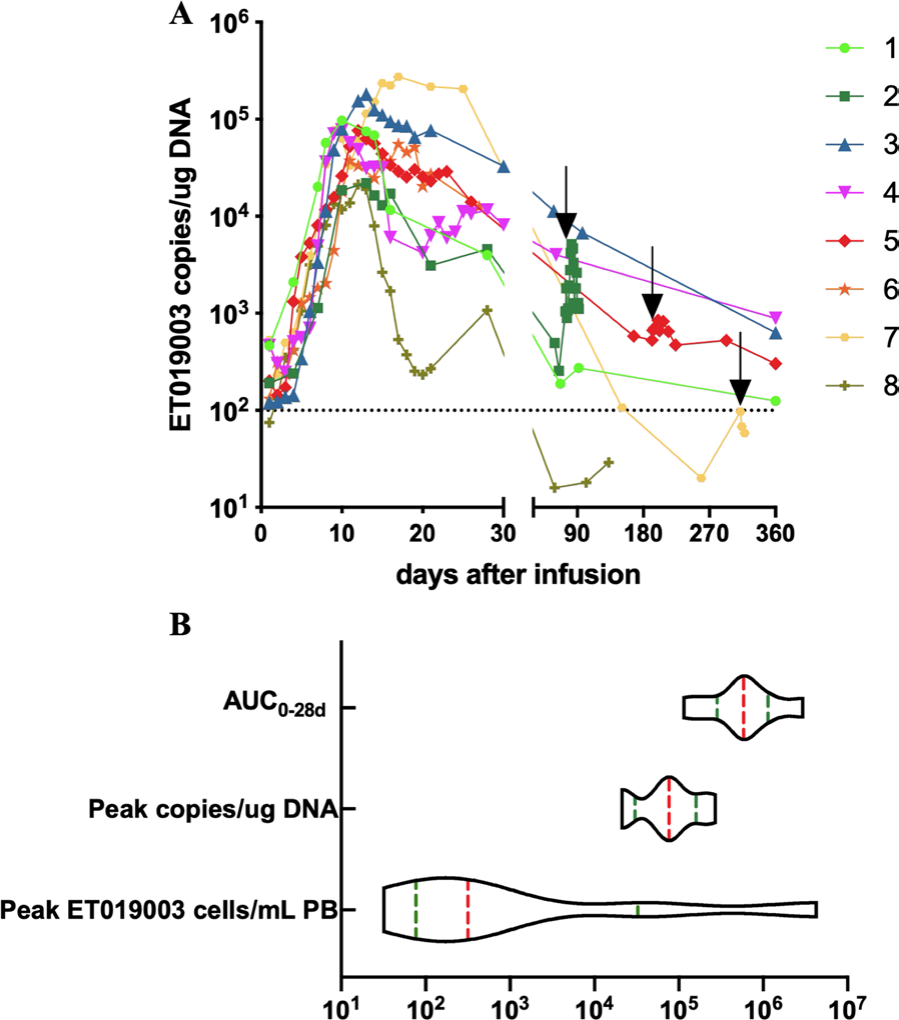
In vivo kinetics of ET019003 cells. **A** ET019003 expansion and persistence were measured as copies per microgram (µg) of the genomic DNA by qPCR in the eight treated patients within 1 year. The detectable threshold was 100 copies per µg of the genomic DNA. The black arrow indicates the second infusion. **B** The violin plot of peak ET019003 cells per milliliter of PB (cells/mL PB) as measured by flow cytometry, peak copies per µg of genomic DNA (copies/µg DNA) as measured by qPCR and area under the curve from 0 to 28 days after infusion (AUC_0–28d_)

ET019003 cells showed peak expansion 9–21 days post infusion (Fig. 5A). ET019003 cells peaked at a median of 318 (range, 32–4,308,109) cells per milliliter of PB as measured by flow cytometry and 76,897 (range, 21,278–273,032) copies per microgram (µg) of genomic DNA as measured by qPCR, respectively. The median area under the curve from 0 to 28 days after infusion (AUC_0–28d_) was 585,493.5 copies per µg × days (Fig. 5B). ET019003 cells continued to be detectable in the PB in 50% of the patients at 12 months. ET019003 expansion was poor in the second infusions.

## Discussion

There is an unmet need for therapies to improve long-term prognosis of patients with RR NHL. CD19-specific CAR-T cell therapy has altered the natural history of RR NHL in comparison with historical controls [5, 6]. Three approved CAR-T cell products in treatment of RR DLBCL attained an ORR of 52–74% and 2-year OS rates of 40.0–50.5% [16, 18, 19]. However, they are also accompanied by treatment-related toxicities, such as CRS and ICANS, which remain the challenges to be overcome. CRS was reported in 42–92% of patients with severe CRS in 2–22%; ICANS occurred in 21–67% of patients with severe ICANS in 10–32% [16, 18, 19]. To generate a safer CD19-specific cell product, some innovative strategies have been explored, including optimizing the affinity of CD19-binding domain [20], modifying the hinge and transmembrane region [21, 22], and utilizing different costimulatory domains [23]. In the study, we creatively constructed a novel anti-CD19 γ/δ TCR, incorporating a fully human CD19-binding domain with the γ/δ TCR-based intracellular domain. ET019003 cells showed comparable antitumor efficacy but notably less cytokine release than CAR-T cells in vitro and in a xenograft mouse model. In the phase 1 trial, ET019003 cells resulted in an ORR of 87.5%, a CR rate of 75%, 3-year OS of 75.0%, 3-year DOR of 71.4%, and a 3-year PFS of 62.5% in patients with RR DLBCL with a median follow-up of 34 months, and importantly, no > grade 1 CRS and only one reversible ICANS were observed. Notably, patient 1 with PCNSL did not experience CRS or ICANS and attained an ongoing CR for over 3 years.

The molecular design of ET019003 has distinct structural features consisting of ET190L1-TCRs and ET190L1-scFv/CD28 co-stimulatory molecules. First, ET190L1-TCR fuses the Fab domain of ET190L1 with the effector domains from γ/δ TCR constant chains, combining the affinity and specificity of antibody recognition with endogenous T cell activation and regulatory pathways. Distinct from conventional TCR-T platforms is the incorporation of an antibody-binding moiety for target recognition, which enables the extension of the TCR platform to non-MHC-restricted targets. Distinct from CAR-T platforms is the γ/δ TCR-based intracellular domain, which has been proven to induce a slower but more protracted cytotoxic response, notably less cytokine release [8, 9]. Moreover, γ/δ TCR subunits can avoid mispairing with endogenous αβ TCRs. Our previous studies have demonstrated that ET190L1-TCR-T cells maintained comparable antitumor potency to ET190L1-CAR-T and CTL019 cells, yet with the reduction in cytokine release and less exhausted phenotype in vitro and in tumor xenograft models [10]. ET190L1-TCR-T cells were tested in another clinical trial (NCT03379493). Second, the parallel-expressed scFv/CD28 co-stimulatory molecule is activated by CD19 to further activate TCR-T cells [11]. In our preclinical evaluation, ET019003 cells showed similar antitumor activity but dramatically less cytokine release to ET190L1-CAR-T cells (Fig. 1). In the phase 1 trial, all CRS was grade 1 and occurred in three (37.5%) patients, and ICANS was observed in one (12.5%) patient. The elevation of serum cytokine levels after ET019003 infusion was almost modest. Collectively, these data indicate that ET019003 cells produce potent antitumor responses without causing the significant elevation of serum cytokine levels that are primarily responsible for CRS and ICANS. With the improved safety profiles of cellular products, outpatient administration and monitoring are being investigated in more clinical studies [16, 18].

Lymphodepletion regimens create a favorable immune environment for transferred T cells and are beneficial to enable their better engraftment, expansion, and persistence [24]. Although the depth of lymphodepletion with one dose of cyclophosphamide 250 mg/m^2^ in our trial was lower than administered before current CAR-T cells, ET019003 cells showed striking expansion and persistence in patients with RR DLBCL. The mean maximal concentration of ET019003 cells in PB was 76,897 copies per µg DNA, and the mean AUC_0–28d_ was 585,493.5 copies per µg × days. ET019003 cells were detectable at 1 year in 50% of patients who have ongoing CR for over 2 years. Unfortunately, correlation analysis was limited by the small sample size. ET019003 cells utilized a fully human anti-CD19 antibody to lessen immunogenic responses that were observed as adverse factors of CAR-T cell kinetics with murine-derived antibodies [25, 26]. Moreover, the intracellular signaling domains, differing in their functional and metabolic profiles, exert an effect on CAR-T cell kinetics [27]. CARs with 4-1BB domain conferred prolonged durability compared with CD28 by tendentious differentiation into central memory phenotype and preventing exhaustion caused by tonic signaling [28]. In our preclinical studies, ET190L1-TCR-T cells with γ/δ TCR intracellular domain were characterized by more naïve/stem-cell like and less exhausted phenotype, which might contribute to favorable expansion and persistence in the clinical setting [10]. However, we failed to characterize ET019003 cells phenotypically and functionally, which was one limitation of the study.

Despite a remarkably high ORR of CD19-specific CAR-T cell therapy in RR NHL, remission is transient in most patients, with a median PFS of 3–6.8 months [16, 18, 19]. Outcomes of NHL patients (*n* = 61) with progressive disease following CD19-specific CAR-T therapy are poor, with a median OS of 5.3 months [29]. Retreatment with a second infusion provides a potential option to improve outcomes for these patients. A second infusion led to a median OS of 9 months in 21 patients with NHL after failure of a first CAR-T infusion [30]. However, retreatment experience was limited. In view of the fully human ET19003 structure, we pioneered a flexible and feasible design to administer the second infusion for risk–benefit tradeoff. If patients did not experience severe AEs within 1 month or reach optimal responses within 3 months, a second infusion was allowed. Fludarabine-cyclophosphamide lymphodepletion before the first CAR-T infusion and a higher second-infusion dose were identified as the modifiable pretreatment factors independently associated with improved CAR-T kinetics and better outcomes of patients after repeated infusions [30]. In our study, all the three retreated patients received fludarabine and cyclophosphamide before the first infusion, but the same dose was used, which might cripple the response. Moreover, ET019003 expansion was poor in the second infusion, partially accounting for the treatment failure. Immune responses to the first ET019003 infusion might result in the rejection of the second infusion, but immunogenicity was not assessed in the study, which was another limitation of the study. Overall, we draw lessons from our small-sample study and do not recommend a second infusion with the identical cell product.

PCNSL is a rare and aggressive form of extranodal NHL in the absence of systemic spread, representing 4% of all intracranial neoplasms [31]. Despite advances in the initial treatment, 36% of patients had a relapse after the frontline treatment, and approximately 10% have primary refractory disease [32]. Prognosis was poor with an estimated OS of 2 months for refractory cases and 3.7 months for relapsed cases [33]. The optimal salvage regimens for patients with RR PCNSL have not been established. Single-cell analyses identify that brain mural cells express CD19 [34], and the pivotal CAR-T trials largely excluded CNSL for concerns of exacerbating potential ICANS. Nevertheless, CD19-specific CAR-T cells administered intravenously are detectable in CSF [35], suggesting that CAR-T cells can penetrate the blood–brain barrier into the CNS to mediate potential antitumor activity. CD19-directed CAR-T cells have induced CR in 50–60% of patients with PCNSL, and more encouragingly, the toxicities were reversible and tolerable, with all CRS of grade 1–2 and sporadic ICANS of grade 3 [36, 37]. In our trial, patient 1 with PCNSL did not experience CRS or ICANS after ET019003 treatment and attained durable CR for over 3 years. ET019003 cells were detectable in both the PB and CSF. Although data presented herein is limited to one patient, the lack of significant CRS and ICANS in this patient is encouraging and lays a foundation for future study of cellular therapies in this patient population. Notably, PCSNL treatment can be associated with late neurotoxicity [7]. In our study, patient 1 experienced viral encephalitis at month 18 and MOG + encephalomyelitis at month 30. Therefore, long-term follow-up is also needed.

## Conclusion

To our knowledge, this is the first-in-human clinical report of CD19-specific γ/δ TCR-T cell therapy in patients with RR DLBCL. The long-term follow-up of greater than 3 years suggest that ET019003 cells have a good acute and long-term safety profile in patients with RR DLBCL. More encouragingly, ET019003 cells can induce rapid response and durable remission for patients with high-risk profiles [19, 38]. In addition, 87.5% of the patients showed symptomatic or imaging improvement within 1 month after infusion, and 62.5% maintained durable CR without further intervention for over 2 years. Our data suggest that ET019003 cells represent a novel and potent therapeutic option for this otherwise challenging patient population. However, our findings are limited by the small sample size, and a recommended phase 2 dose was not identified. Further larger and multicenter trials are needed to verify the long-term safety and efficacy of CD19-specific γ/δ TCR-T cells in RR DLBCL. We hope our preliminary results will shed some light on novel strategies of cellular therapies.

## Supplementary Information

**Additional file 1**: Supplementary methods, results, table, and figures.

## Data Availability

The original contributions presented in the study are included in the article or uploaded as Additional file 1.

## Abbreviations

AEs: Adverse events
CAR: Chimeric antigen receptor
CRS: Cytokine release syndrome
CR: Complete response
CI: Confidence interval
CSF: Cerebrospinal fluid
DLBCL: Diffuse large B-cell lymphoma
DLTs: Dose-limiting toxicities
DOR: Duration of response
GCB: Germinal center B-cell
ICANS: Immune cell associated neurotoxicity syndrome
IL: Interleukin
NHL: Non-Hodgkin lymphoma
OS: Overall survival
ORR: Overall response rate
PCNSL: Primary central nervous system lymphoma
PFS: Progression-free survival
PB: Peripheral blood
PR: Partial response
PET-CT: Positron emission tomography-computed tomography
RR: Relapsed or refractory
scFv: Single-chain fragment variable
TCR: T cell receptor
µg: Microgram
