# Supplementary material for: Novel CD19-specific γ/δ TCR-T cells in relapsed or refractory diffuse large B-cell lymphoma
Source: J Hematol Oncol. 2023 Jan 21;16:5. doi: 10.1186/s13045-023-01402-y (PMC9862812; doi:10.1186/s13045-023-01402-y)
Supplement: Supplementary file 1 — Additional file 1: Supplementary methods, results, table, and figures. [file 13045_2023_1402_MOESM1_ESM.pdf]

Additional file 1

Novel CD19-specific γ/δ TCR-T cells in Relapsed or Refractory Diffuse Large B-Cell Lymphoma

Authors: Chenggong Li[1,2]*, Fen Zhou[2,3]*, Jing Wang[4], Qi Chang[5], Mengyi Du[1,2], Wenjing Luo[1,2], Yinqiang Zhang[1,2], Jia Xu[1,2], Lu Tang[1,2], Huiwen Jiang[1,2], Lin Liu[1,2], Haiming Kou[1,2], Cong Lu[1,2], Danying Liao[1,2], Jianghua Wu[1,2], Qiuzhe Wei[1,2], Sha Ke[1,2], Jun Deng[1,2], Cheng Liu[5], Heng Mei[1,2#], and Yu Hu[1,2#]

Supplemental methods 2-4

Supplemental results 5

Table S1 6

Figure S1-S6 7-12

Reference 13

Supplemental methods

Preclinical studies

Raji and Nalm-6 cells were obtained from ATCC. Raji-luciferase cell line was purchased from Comparative Biosciences, Inc. (Sunnyvale, CA). Generation of ET019L1-CAR and ET019L1-CAR-T cells was detailed previously [1]. ET019003 and ET019L1-CAR-T cells were cultured with $CD19^+$ Raji and Nalm-6 cells at an effector-to-target ratio of 2:1 for 16–24 hours to test cytotoxicity. Cytokine release in culture supernatants and mouse serum was measured using the Bio-plex Pro Human Cytokine 8-plex assay (BioRad, California, USA). Animal experiments were conducted and approved by Murigenics, Inc (California, USA). Female NSG mice (The Jackson Laboratory) aged 6–8 weeks were used. Mice were inoculated with 1×10^6 Raji-luciferase cells by tail vein injection 5 days in advance, followed by administration of 1.0×10^6 mock T cells, ET019L1-CAR-T cells, and ET019003 cells. Bioluminescence imaging was performed using the IVIS Imaging System with the Living Image software (PerkinElmer, Massachusetts, USA). The mice were observed for mortality up to 60 days after T cell administration. The survival was graphically represented as Kaplan-Meier curves and analyzed by using the log-rank test.

ET019003 cell production and monitoring

Patients underwent leukapheresis to obtain (5–10) $\times10^9$ autologous peripheral blood mononuclear cells, and T cells were isolated using CD3-negative microbeads (Miltenyi Biotec, Germary) and stimulated with CD3/CD28 dynabeads (Thermo Fisher Scientific, Massachusetts, USA) for 24 hours. Activated T cells were transduced with ET019003 lentivirus at a multiple of infection of 5 and cultured as previously described [1-3]. Nine days after the initiation of production, ET019003 cell product was tested for identity, potency, sterility, and adventitious agents. After meeting the acceptance criteria as previously given [4], ET019003 cell product was shipped back to the clinical ward using a validated fresh or cryopreserved shipper for infusion. The transduction efficiency of TCR+T cells was determined by flow cytometry using a PE-labeled ITET0190 antibody (Eureka Therapeutics, California, USA) and normalized with the fluorescence minus one control. Primary antibodies for phenotypical identification were obtained from BioLegend

(California, USA): FITC anti-CD3 (clone UCHT1), APC anti-CD4 (clone A161A1), and PerCP-Cy5.5 anti-CD8 (clone HIT8a). All antibodies used in this study were titrated before use.

ET019003 cells in peripheral blood (PB) and cerebrospinal fluid were detected by flow cytometry and quantitative PCR. For flow quantitation, PB samples from patients before or after ET019003 cell infusion were processed within 24-48 hours. All samples were stored at 4°C before the experiments. Fresh PB samples were processed with red blood cells lysis and PBS washing, followed by staining with ITET0190 and anti-CD3 antibody. Within the lymphocyte gate in clinical samples, at least 10,000 events were acquired by BD FACSVia^{TM} flow cytometry. Voltage is the factory setting. Quality control using QC particles was routinely conducted. Data were analyzed by BD Accuri C6 plus Software.

Genomic DNA was isolated from fresh blood samples obtained before infusion and at serial time points after ET019003 infusion using AllPrep DNA/RNA Mini Kit (Qiagen, Germany). The primers and probe for ET019003 cells were designed to target wood-chuck hepatitis virus post-transcriptional regulatory element, a post-transcriptional regulatory element present in the lentiviral vector. The sequence of primes was as follows: the upstream prime, GCATTGCCACCACCTGTCA; the downstream prime, TCCGCCGTGGCAATAGG; the probe, FAM-CTTTCCGGGACTTTCG-MGB (Synbio Tech, Suzhou, China). Vector plasmids with known-copy number of integrated TCR were diluted by gradient concentration and used for the standard curve. All samples were measured three times using Applied Biosystems^{TM} 7500. Results were reported as copies per microgram of the genomic DNA, with a detection limit of 100 copies per microgram of the genomic DNA.

Inclusion criteria

Eligible patients must have (1) histologically confirmed DLBCL; (2) documented CD19 expression on malignant cells by immunohistochemistry; (3) refractory disease as defined in the SCHOLAR-1 study, or recurrent disease within 6 months or at least 2 times after CR; (4) prior therapy including an anti-CD20 monoclonal antibody and an anthracycline; (5) at least one measurable lesion according to the Lugano criteria [5]; (6) at least 2 weeks since prior radiation or systemic therapy at the time of

leukapheresis; and (7) the toxicities related to previous treatments returned to ≤ grade 1 (except for low-grade toxicity such as alopecia). Eligible patients were also aged 18-75 years, with ECOG performance status ≤ 2, and estimated survival time ≥ 3 months. Patients must have had adequate cardiac, hepatic, pulmonary, and renal functions defined as left ventricular ejection fraction ≥ 50%, serum alanine aminotransferase and aspartate aminotransferase ≤ 3 times of the upper limit of normal (ULN), total bilirubin ≤ 2 times of ULN, indoor oxygen saturation ≥ 95%, and serum creatinine of < 220μmol/L. Given the preclinical cytokine profiles of ET019003 cells, patients with primary or secondary CNS lymphoma were eligible, and ECOG performance status > 2 due to CNS involvement were acceptable.

Supplemental results

Diagnosis and treatment of two late adverse events in patient 1

At month 18 after infusion, patient 1 suddenly had psychosis, accompanied by irritability and nonsense, and did not cooperate with the physical examination. Then, the patient experienced fever, with elevated serum high sensitivity C-reactive protein (8 times of the normal upper limit). The cerebral plain and enhanced magnetic resonance imaging did not found any residual or new lesions. Cerebrospinal fluid (CSF) examination showed slight elevation of the intracranial pressure (190 mmHg), chloride, protein, and sugar. Total cells and leukocytes in CSF were normal, and exfoliative cytology did not find any abnormalities. ET019003 cells were undetectable in the peripheral blood (PB) and CSF. CSF smear combined with bacterial and fungal culture excluded infections with fungi, acid-fast bacteria, and *Cryptococcus neoformans*. Tuberculosis by Xpert was negative. The blood cultures of fungi, aerobic and anaerobic bacteria was negative, and EBV-DNA and CMV-DNA quantification in blood was negative. Endocrine testing excluded thyroid, adrenal, and pituitary dysfunctions. Based on the above clinical evidence, the patient was empirically administered with ganciclovir 0.25g q12h. The patient's symptoms improved and gradually returned to normal. Together, the patient was diagnosed with viral encephalitis, undefined virus.

At month 30 after infusion, patient 1 had psychosis, accompanied by nonsense without obvious inducement again, and did not cooperate with the physical examination. The imaging examination did not detect any residual or new lesions. Routine, biochemical, and multicolor flow cytometric assessment, and microbiological examination of CSF were negative. The blood routine, C-reactive protein, and microbiological examinations were also normal, but the serum demyelination test showed MOG positivity. ET019003 cells were undetectable in the PB and CSF at that time. Accordingly, the patient was diagnosed with MOG+ encephalomyelitis and was administered with high-dose corticosteroids.

Table S1. ET019003-T Cell Product Characteristics

Patient ID	ET019003+/CD3 + cells on Day 9	CD4+ in ET019003-T	CD8+ in ET019003-T	Days from initiation of manufacture to infusion	Dose of infused ET019003-T cells	Fresh/Cryopreserved
1	60.8%	57.8%	41.2%	13	$2x10^6$/kg	fresh
2-1st	59.6%	79.8%	18.5%	11	$2x10^6$/kg	fresh
2-2nd	64.0%	91.8%	8.3%	13	$2x10^6$/kg	fresh
3	65.4%	46.5%	50.4%	12	$2x10^6$/kg	fresh
4	61.1%	71.1%	29.8%	12	$2x10^6$/kg	fresh
5-1st	75.90%	79.40%	19.50%	25	$2x10^6$/kg	cryopreserved
5-2nd				217	$2x10^6$/kg	cryopreserved
6	44.90%	43.20%	54.40%	12	$2x10^6$/kg	fresh
7-1st	52.40%	51.30%	44.30%	11	$4x10^6$/kg	fresh
7-2nd	50.50%	61.10%	31.30%	20	$4x10^6$/kg	cryopreserved
8	60.60%	31.80%	51.60%	13	$4x10^6$/kg	fresh
Mean	59.52%	61.38%	34.93%	14*		

*Time of patient 5 in the second infusion with the cryopreserved ET019003 cells was not included in the calculation.

Supplemental Figures

Figure S1. Study CONSORT diagram.

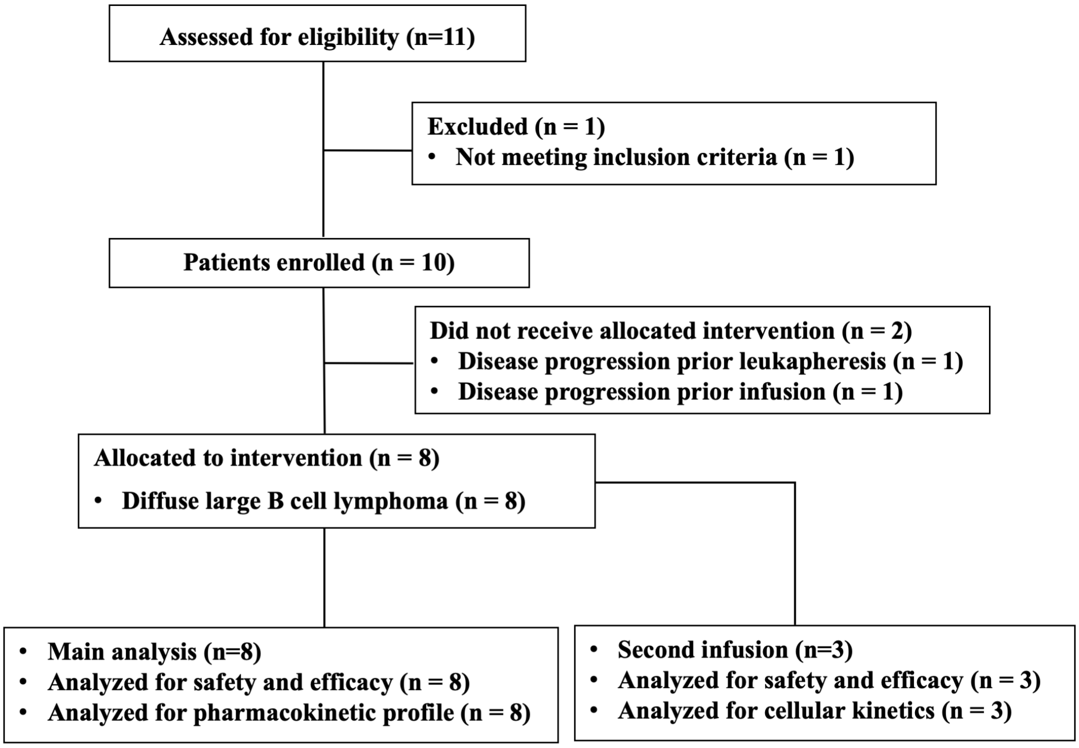

Figure S2. Changes of blood liver, kidney, and nutritional markers during ET019003 cell therapy. ALT: alanine aminotransferase; AST: aspartate aminotransferase; ALP: alkaline phosphatase; LDH: lactate dehydrogenase; LNL: lower limit of normal value; UNL: upper limit of normal value.

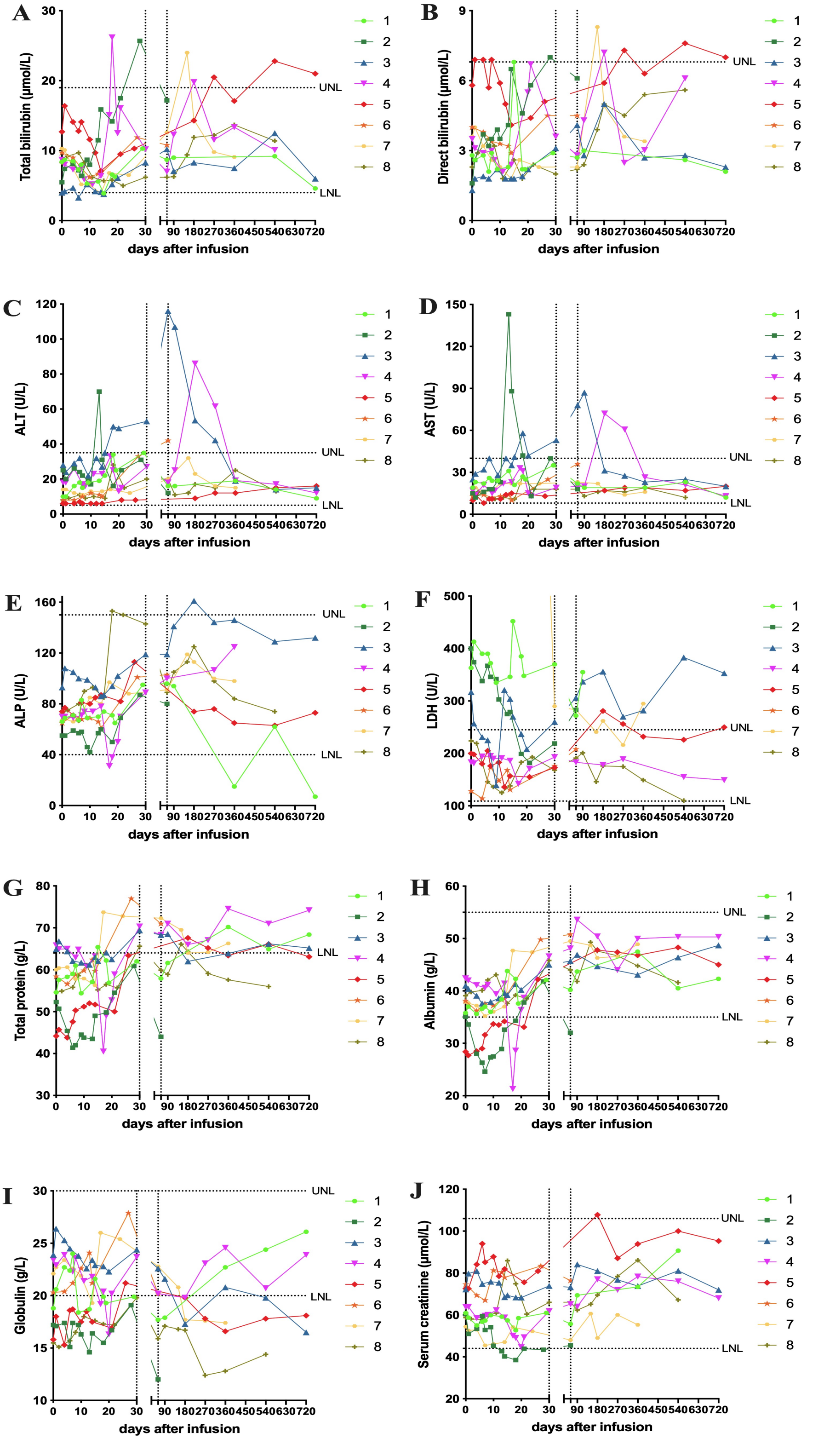

Figure S3. Hematological changes within 2 years after ET019003 infusion. (a-f) Changes in white blood cells (WBC) count, hemoglobin concentration, platelet count, neutrophil count, lymphocyte count, and monocyte count in each patient within 2 years. LNL, lower limit of normal value; grade 2 according to CTCAE v5.0. **(g)** Effects of the preconditioning regimens, fludarabine and cyclophosphamide (FC), on the hemocytes. The hemocyte counts were chosen on day -5 and day 0 before infusion for the paired t test. The patients did not receive blood transfusion or any colony-stimulating factor during the period. Ns, not significant.

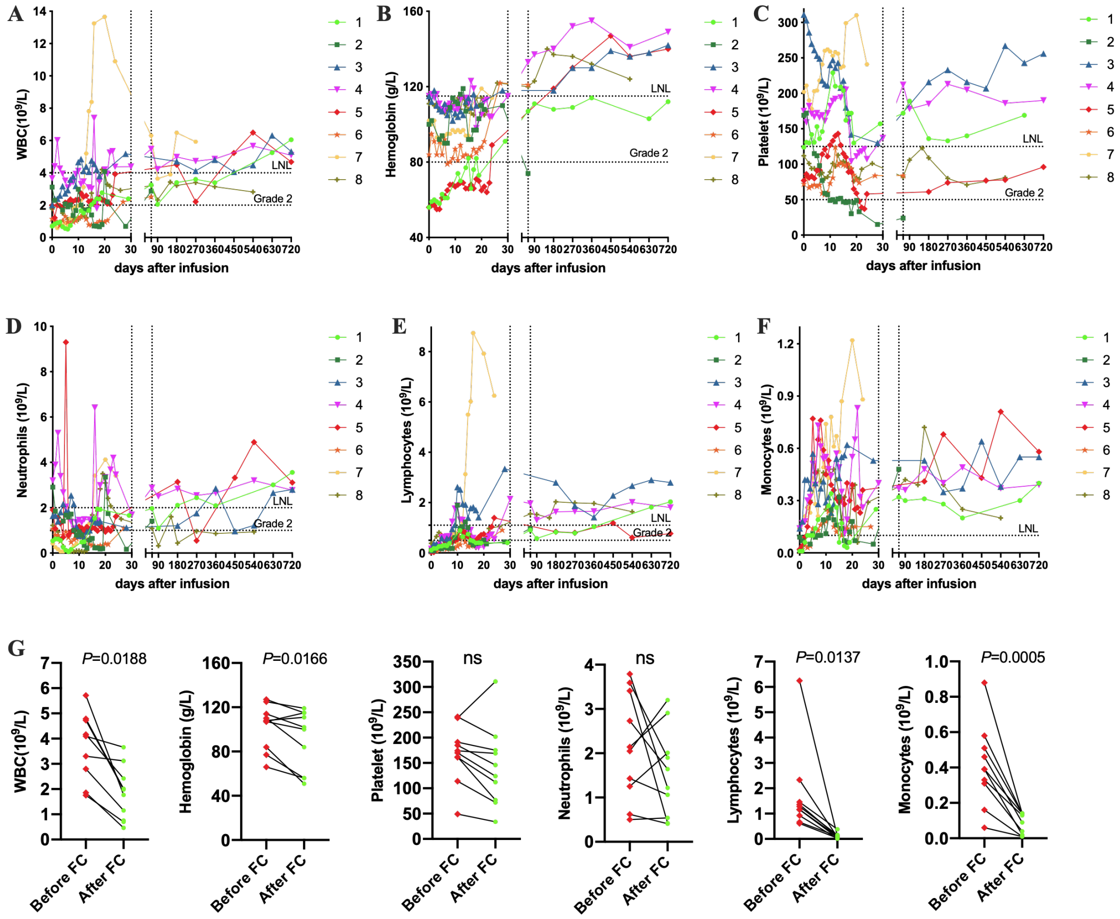

Figure S4. Changes in lymphocyte subsets and serum immunoglobulins after ET019003 infusion. (a-f) Changes in B cells, NK cells, $CD3^{+}$ T, $CD4^{+}$ T, $CD8^{+}$ T, and the ratio of CD4 to CD8 in peripheral blood within 1 month after ET019003 infusion. Wilcoxon matched-pairs rank test was used because the data were not normally distributed. Ns, not significant. **(g-i)** Changes in serum immunoglobulin (Ig)G, IgA, and IgM in individuals within 2 years. LNL, lower limit of normal value; UNL, upper limit of normal value; FC, fludarabine and cyclophosphamide; D0, the day of ET019003 infusion; D, day; M, month.

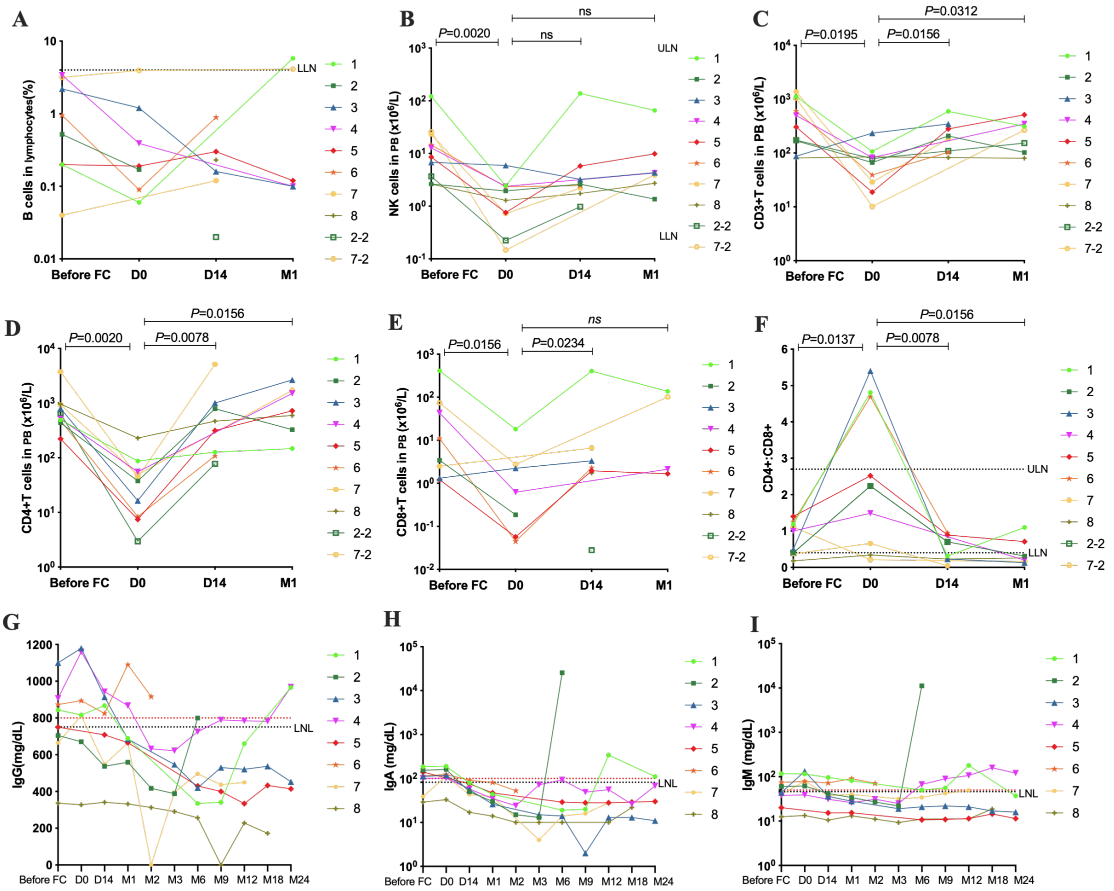

Figure S5. Clinical responses of ET019003 cells. (a) Abdominal enhanced computed tomography (CT) scans of patient 2 before treatment, on day 15 and day 63 after the first infusion, and on day 14 after the second infusion. **(b)** Pelvic enhancement CT and cervical enhanced magnetic resonance imaging (MRI) scans of patient 4 before treatment, on day 24 and day 61 after infusion. **(c)** Abdominal enhanced CT scans of patient 5 before treatment, on day 14 after infusion, and at the last follow-up. **(d)** Abdominal enhanced CT scans of patient 6 before treatment, on day 14 and day 28 after infusion. **(e)** Positron emission tomography-computed tomography (PET-CT) scans of patient 7 on day 262 after the first infusion and on day 30 after the second infusion. **(f)** CT and PET-CT scans of patient 8 before treatment, on day 24 after infusion and at the last follow-up.

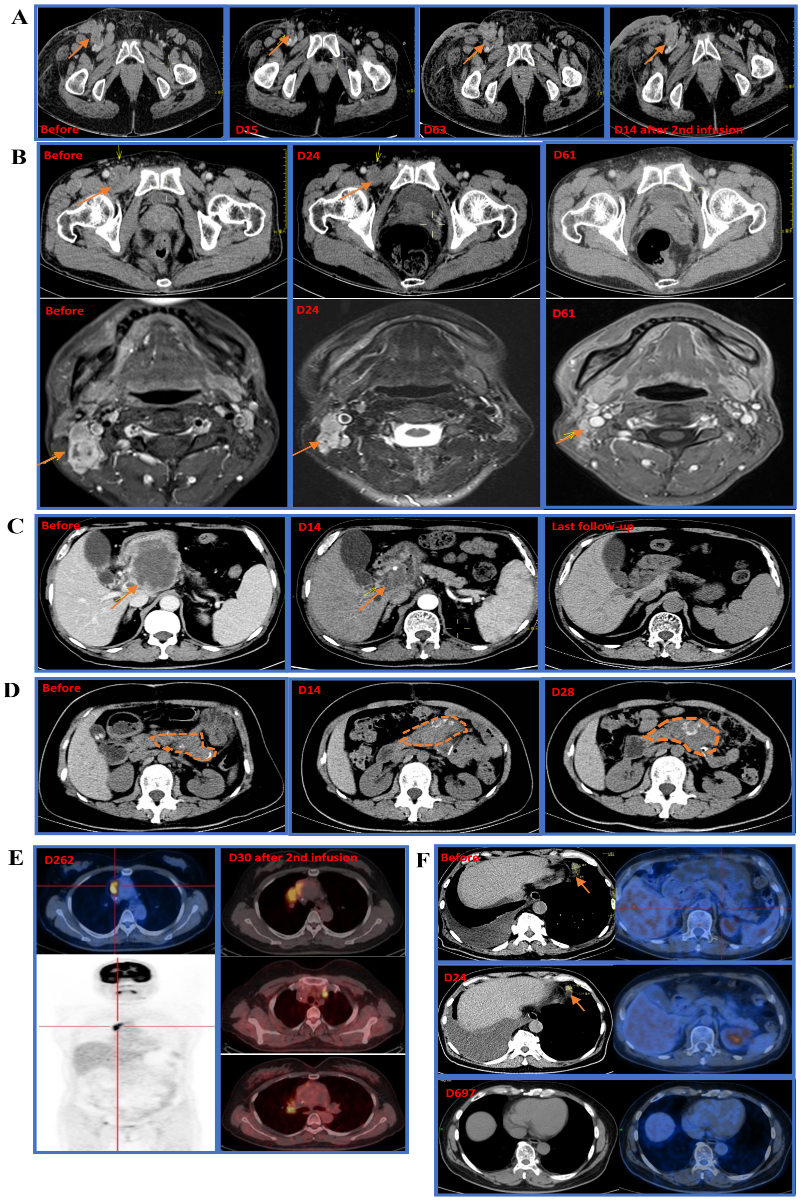

Figure S6. Changes of hematological, liver, kidney, and nutritional markers after the second ET019003 infusion. WBC: white blood cell; ALT: alanine aminotransferase; AST: aspartate aminotransferase; ALP: alkaline phosphatase; LDH: lactate dehydrogenase; LNL: lower limit of normal value; UNL: upper limit of normal value; Grade 2 according to CTCAE v5.0.

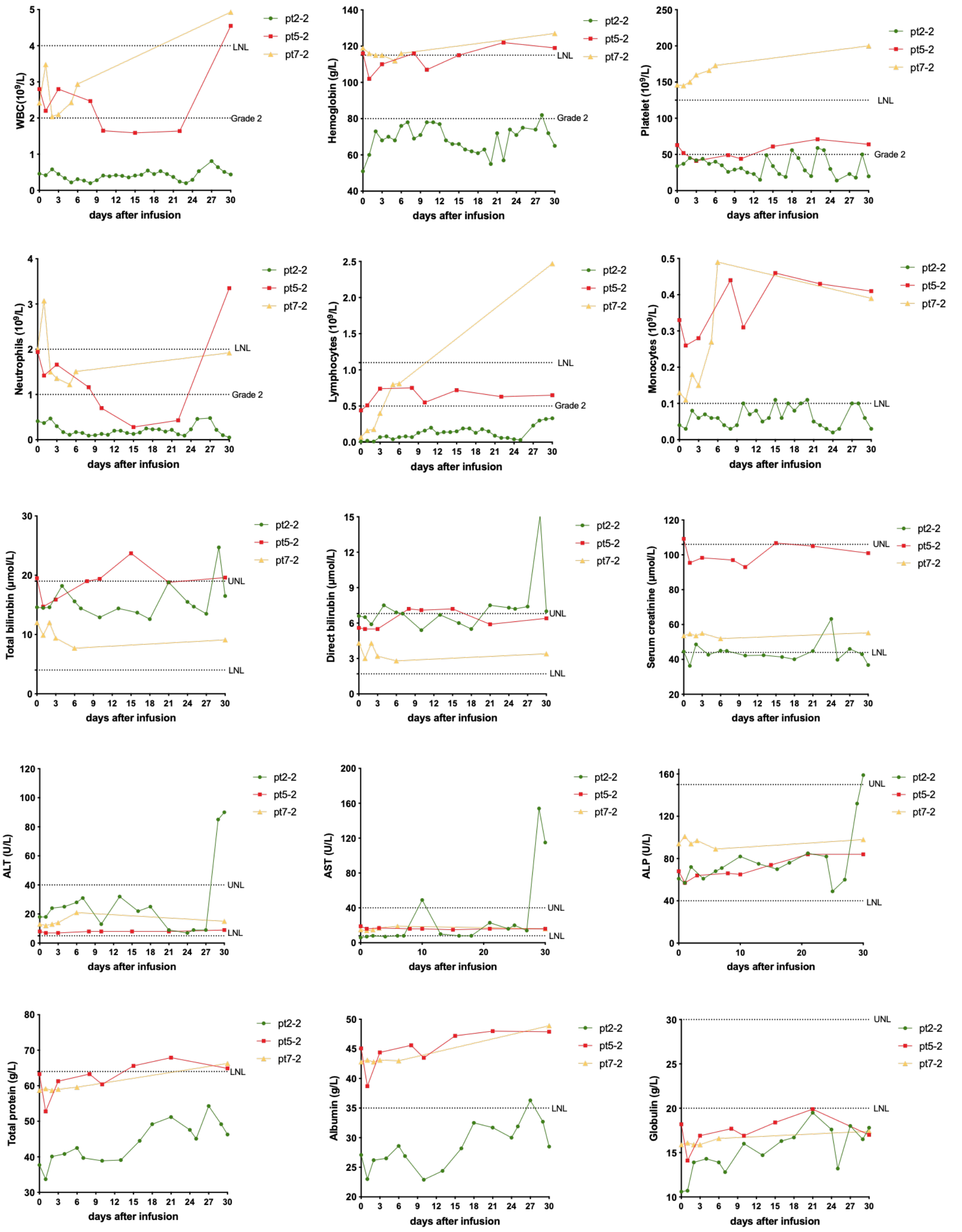

Reference

1. Xu Y, Yang Z, Horan LH, et al. A novel antibody-TCR (AbTCR) platform combines Fab-based antigen recognition with gamma/delta-TCR signaling to facilitate T-cell cytotoxicity with low cytokine release. Cell Discov. 2018;4:62.
2. Liu H, Xu Y, Xiang J, et al. Targeting Alpha-Fetoprotein (AFP)-MHC Complex with CAR T-Cell Therapy for Liver Cancer. Clin Cancer Res. 2017;23(2):478-488.
3. Liu C, Liu H, Dasgupta M, et al. Validation and promise of a TCR mimic antibody for cancer immunotherapy of hepatocellular carcinoma. Sci Rep. 2022;12(1):12068.
4. Ali SA, Shi V, Maric I, et al. T cells expressing an anti-B-cell maturation antigen chimeric antigen receptor cause remissions of multiple myeloma. Blood. 2016;128(13):1688-1700.
5. Cheson BD, Fisher RI, Barrington SF, et al. Recommendations for initial evaluation, staging, and response assessment of Hodgkin and non-Hodgkin lymphoma: the Lugano classification. J Clin Oncol. 2014;32(27):3059-3068.
